# Knockdown of TRPM7 attenuates apoptosis and inflammation in neonatal necrotizing enterocolitis model cell IEC-6 via modulating TLR4/NF-κB and MEK/ERK pathways

**DOI:** 10.22038/IJBMS.2022.62113.13742

**Published:** 2022-08

**Authors:** Lu An, Juan Li, Bing Liu, Junpeng Hui, Qiang Zhang, Xin Zhang, Qi Wang

**Affiliations:** 1 Department of Pathology, Xi’an Children’s Hospital, Xi’an 710003, Shaanxi Province, China; 2 Department of Neonatal Surgery, Xi’an Children’s Hospital, Xi’an 710003, Shaanxi Province, China

**Keywords:** Apoptosis, Inflammation, Necrotizing enterocolitis, TRPM7, Viability

## Abstract

**Objective(s):**

Neonatal necrotizing enterocolitis (NEC) is the most common gastrointestinal critical illness in neonatal infants. TRPM7 reportedly plays a role in human inflammatory bowel disease (IBD) and colorectal cancer, but the role of TRPM7 in the pathogenesis of NEC remains vague.

**Materials and Methods:**

The expression of TRPM7 was determined in intestinal tissues of NEC patients and lipopolysaccharide (LPS)-induced IEC-6 cells. Subsequently, a loss-of-function assay was performed to assess the effects of TRPM7 on cell apoptosis and inflammatory response in IEC-6 cells after LPS induction. Furthermore, the modulation of TRPM7 on TLR4/NF-κB and MEK/ERK signaling pathways was validated.

**Results:**

The expression of TRPM7 was higher in the intestinal tissues of NEC patients compared with the normal human intestinal tissues. Moreover, the expression level of TRPM7 was elevated in LPS stimulation IEC-6 cells. Knockdown of TRPM7 enhanced cell viability and suppressed apoptosis, accompanied by the decreased Bax/Bcl-1 ratio and cleaved-caspase3 expression in LPS-induced IEC-6 cells. Additionally, TRPM7 silencing attenuated LPS-induced expressions and secretions of proinflammatory cytokines. Mechanistically, TRPM7 knockdown inhibited the TLR4/NF-κB activation, while enhancing the MEK/ERK activation in LPS-treated IEC-6 cells. Overexpression of TLR4 or inhibition of MEK attenuated the inhibitory effects of TRPM7 knockdown on LPS-induced apoptosis and inflammation in IEC-6 cells.

**Conclusion:**

Knockdown of TRPM7 attenuated LPS-induced IEC-6 cell apoptosis and inflammation by modulating TLR4/NF-κB and MEK/ERK pathways.

## Introduction

Neonatal necrotizing enterocolitis (NEC) is an inflammatory bowel necrosis commonly in premature and low birth weight infants (1, 2). Clinically, it is characterized by vomiting, abdominal distension and bloody stools, and complicated by severe diseases (2-4). Current research believes that the onset of NEC is the result of a combination of multiple factors caused by an imbalance of anti-inflammatory and pro-inflammatory factors (2). During this process, a large number of bacterial endotoxins such as lipopolysaccharide (LPS) are released, which further promotes the development of inflammation (5, 6). However, the early symptoms of NEC are not specific. Many infants with NEC did not receive adequate therapy, leading to further injuries to the intestine and other organs (7). Therefore, there is an urgent need to understand the pathogenesis of NEC and find specific prevention and treatment methods.

Transient receptor potential melastatin 7 (TRPM7) is a unique bifunctional protein with dual domains of ion channel and protein kinase, which is widely distributed in a variety of tissues and organs (8, 9). By regulating the potential of the cell membrane, TRPM7 participates in a variety of biological processes (10, 11). It has been shown that TRPM7 regulated intestinal motility in mice by inhibiting pacemaker potentials in interstitial cells of Cajal (12, 13). In addition, a recent study showed that TRPM7 was involved in the pathophysiology of human inflammatory bowel disease (IBD) and colorectal cancer (14). Importantly, abnormal expression of TRPM7 was found in various inflammatory diseases (9, 15, 16). However, the role and potential regulatory mechanism of TRPM7 in NEC remain unknown.

Studies have found that expression of TLR4 in the small intestine of NEC patients and model mice was significantly increased (17, 18). It is reported that TLR4 is the main receptor of LPS; LPS-induced TLR4 activation caused damage to the intestinal barrier. Nuclear factor-κB (NF-κB) is an effector molecule of the TLR4 signaling pathway and induces massive release of inflammatory mediators, causing local intestinal mucosal epithelial cell apoptosis and damaging intestinal tissues and organs (6, 19). Besides, MEK activates serine/threonine protein kinase ERK1/2 phosphorylation and mediates a variety of transcription factors ultimately promoting cell proliferation and blocking cell apoptosis (20, 21). Importantly, the MEK/ERK pathway is obviously activated in colitis (22). However, inhibition of the MEK/ERK pathway promotes the occurrence and development of colitis (23), which might be because the MEK/ERK pathway is a repair mechanism for intestinal epithelial cells.

In our study, we established an LPS-induced *in* *vitro* NEC model. The expression of TRPM7 was detected and the effects of TRPM7 on cell apoptosis and inflammation in LPS-induced IEC-6 cells were validated. Moreover, we explored the potential regulatory mechanism of TRPM7 in LPS-induced IEC-6 cells. Our study aimed to provide a novel molecular biomarker for the prevention and treatment of NEC.

## Materials and Methods

***Materials***

Human intestinal epithelial cell line IEC-6 was obtained from American Type Culture Collection (USA). Dulbecco’s Modified Eagle’s Medium, fetal bovine serum (FBS) and phosphate-buffered saline (PBS) buffer, Lipofectamine 3000 kit, and TRIzol reagent were purchased from ThermoFisher Scientific lnc. (USA). LPS was obtained from Nanjing Jiancheng Bioengineering Institute (China). The small interfering RNAs and overexpression plasmids were synthesized from Generay Biotech (China). CCK8 kit was obtained from Dojindo (Japan). Annexin V-FITC/PI apoptosis detection kit was purchased from BioVision (USA). Prime Script^TM^ RT reagent kit and SYBR Premix Ex Taq^TM^ were obtained from Takara (Japan). The PCR primers were obtained from Shanghai Genechem Co., Ltd (China). ELISA kits were obtained from R&D Systems Inc. (USA). BCA kit was purchased from Wuhan Doctor Bioengineering Co., Ltd (China). All antibodies were purchased from Abcam (USA) (TRPM7; Bcl-2; Bax; Cleaved Caspase 3; TLR4; p65; MEK; ERK; GAPDH) and Santa Cruz Biotechnology (USA) (p-p65; p-MEK; p-ERK). The enhanced ECL kit was purchased from Millipore (USA). The MEK pathway inhibitor, PD98059, was obtained from Sigma-Aldrich (USA). A flow cytometer was obtained from BD Bioscience (USA). The microplate reader was purchased from Multiskan Spectrum (USA). SpectraMax M5 fluorimeter was obtained from Molecular Devices (USA).

***Tissue samples***

The NEC intestinal tissue specimens (n=14) and their adjacent normal tissue specimens (n=14) were collected from neonates and infants undergoing bowel resection from the Affiliated Children Hospital of Xi’an Jiaotong University Hospital. The study was conducted in accordance with the Declaration of Helsinki. The acquisition of the tissue samples was approved by the Affiliated Children Hospital of Xi’an Jiaotong University (20220002). We obtained written informed consent from guardians before the study.

***Cell culture***

Human intestinal epithelial cell line IEC-6 was cultured in Dulbecco’s Modified Eagle’s Medium with 10% fetal bovine serum at 37 °C in a humidified atmosphere containing 5% CO_2_. We treated IEC-6 cells with 100 μg/ml LPS for 3 hr as an *in vitro* NEC cell model according to the method of Yuan *et al* (24).

***Cell transfection***

The small interfering RNAs of TRPM7 (siTRPM7-1 and siTRPM7-2) and overexpression plasmids of TLR4 (pcDNA-TLR4) were constructed. IEC-6 cells were transfected with siTRPM7s or/and pcDNA-TLR4 by Lipofectamine 3000 Kit for 48 hr before LPS treatment.

***Cell viability assay***

The cell viability of IEC-6 cells was measured by Cell Counting Kit-8. IEC-6 cells were seeded into 96-well plates (1×10^4^ cells per well) for 24 hr, then treated with 100 μg/ml LPS for 3 hr. Subsequently, cells were incubated with 10 µl CCK-8 at 37 °C for 2 hr. The absorbance at 450 nm was measured using a SpectraMax M5 fluorimeter.

***Apoptosis assay***

The apoptotic rate of IEC-6 cells was quantitatively analyzed using Annexin V-FITC/PI apoptosis detection kit. Briefly, IEC-6 cells (5×10^5^ cells per well) were washed twice with cold PBS and re-suspended. Then, 10 μl Annexin V-FITC and PI were added to cell samples; and the samples were incubated at room temperature in the dark for 1 hr. At last, apoptotic cells were measured by a flow cytometer.

***Quantitative real-time polymerase chain reaction (qRT-PCR)***

The total RNA of tissues or IEC-6 cells was extracted by TRIzol Reagent. Prime Script^TM^ RT reagent Kit and SYBR Premix Ex Taq^TM^ were used to measure the mRNAs expression, according to the manufacturer’s protocol. GAPDH served as an internal control. The relative expression of the target gene was calculated according to a relative quantification (2−^ΔΔCt^) method and normalized. The sequences of primers are: TRPM7: Forward 5’-CCATACCATATTCTCCAAGGTTCC-3’, Reverse 5’-CAT

TCCTCTTCAGATCTGGAAGTT-3’; proinflammatory cytokines, tumor necrosis factor-α (TNF-α): Forward 5’-TTCGAGTGACAAGCCTGTAGC-3’, Reverse 5’-AGATT

GACCTCAGCGCTGAGT-3’; , Interleukin-1β (IL-1β): Forward 5’-AATCTCACAGCACATCAA-3’, Reverse 5’-AGCCCATACTTTAGGAAGACA-3’; Interleukin-6 (IL-6): Forward 5’- GAGGATACCACTCCCAACAGACC-3’, Reverse 5’- AAGTGCATCATCGTTGTTCATACA-3’; GAPDH: Forward 5’- TGACTTCAACAGCGACACCCA-3’, Reverse 5’-CACCCTGTTGCTGTAGCCAAA-3’.

***Western blotting***

The total proteins were extracted from tissues or IEC-6 cells and protein concentrations were detected according to instructions of the BCA kit. Protein samples were loaded into SDS-PAGE and transferred to PVDF membranes. After blocking with 5% non-fat milk, the primary antibodies (anti-TRPM7 1:500; anti-Bcl-2, 1:800; anti-Bax, 1:1000; anti-Cleaved Caspase 3 1:1000; anti-TLR4 1:1000; anti-NF-κBp65 1:1000; anti-MEK 1:1000; anti-ERK 1:1000; anti-GAPDH 1:3000; anti-p-p65 1:1000; anti-p-MEK 1:1000; anti-p-ERK 1:1000) were cultured at 4 °C overnight. Then, the membranes were incubated at room temperature with horseradish peroxidase combined with secondary antibody for 1 hr. The bands were detected by an enhanced ECL kit. Image J software was used to measure the density of the membrane quantitatively.

***Enzyme-linked immunosorbent assays (ELISA)***

TNF-α, IL-1β, and IL-6 secretion in the culture medium of IEC-6 cells after LPS induction and the corresponding vector transfection were measured using an ELISA kit, according to the protocols of the manufacturer. All samples were assessed by a microplate reader.

***Statistical analysis***

All experiments were repeated more than three times. Statistical analysis was done using SPSS 22.0 software. The results are presented as the mean ± SEM. Student’s t-test or one-way analysis of variance was used to carry out statistical analysis. The result value of *P*<0.05 was represented as statistically significant.

## Results

***TRPM7 was up-regulated in NEC tissues and LPS-induced IEC-6 cells***

**Figure 1:**
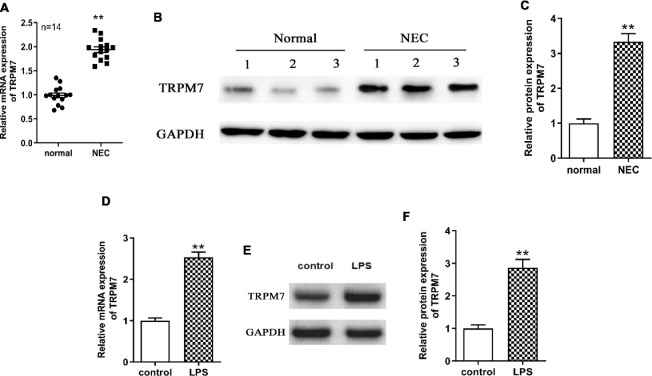
TRPM7 was up-regulated in necrotizing enterocolitis (NEC) tissues and lipopolysaccharide (LPS)-induced IEC-6 cells. (A) qRT-PCR was performed to determine the mRNA expression levels of TRPM7 in NEC intestinal tissues (n=14) and adjacent normal tissues (n=14). ** means * P*<0.01 vs normal tissues. (B-C) Protein expression level of TRPM7 in NEC intestinal tissues (n=3) and normal tissues (n=3) was detected by Western blotting. GAPDH as an internal reference. ** means * P*<0.01 vs normal tissues. (D-F) IEC-6 cells were stimulated with 100 μg/ml LPS for 3 hr to establish an in vitro NEC cell model (LPS group), and cells treated with an equal amount of culture medium were used as controls (control group). mRNA and protein expression levels of TRPM7 in the LPS group and control group were determined using qRT-PCR and western blotting. ** means* P*< 0.01 vs the control group

To explore the potential involvement of TRPM7 in the pathogenesis of NEC, we detected the TRPM7 expression in intestinal tissues of NEC patients. As shown in Figures 1A-1C, the mRNA and protein expressions of TRPM7 were significantly increased in NEC intestinal tissues compared with normal intestinal tissues (*P*<0.01). *In vitro*, the results of qRT-PCR and western blotting determined that TRPM7 mRNA and protein expressions of intestinal epithelial cells IEC-6 were up-regulated after LPS stimulation (Figures 1D-1E) (*P*<0.01).

***Knockdown of TRPM7 restored viability and inhibited apoptosis in LPS-induced IEC-6 cells***

**Figure 2:**
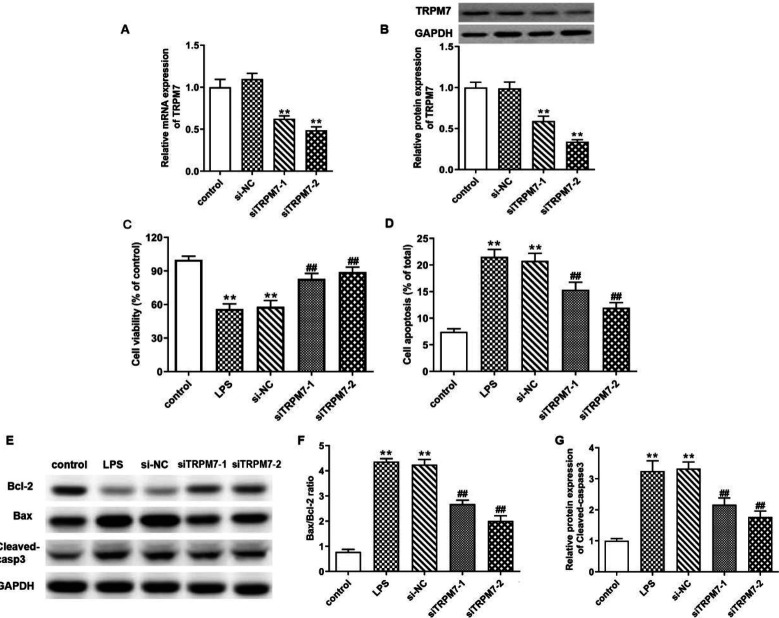
Knockdown of TRPM7 restored viability and inhibited apoptosis in lipopolysaccharide (LPS)-induced IEC-6 cells. (A-B) IEC-6 cells were transfected with the synthetic TRPM7 siRNA1 and siRNA2 or their negative control (si-NC) for 48 hr before LPS treatment. QRT-PCR and western blotting were performed to determine the mRNA and protein expression levels of TRPM7 in LPS-induced IEC-6 cells after siTRPM7 transfection, non-transfected IEC-6 cells performed as a control. (C) CCK-8 assay was performed to determine the effect of siTRPM7 on LPS-induced IEC-6 viability. (D) Flow cytometry analysis was performed to evaluate the effect of TRPM7 siRNAs on LPS-induced IEC-6 apoptosis. (E-G) Western blotting analyzed the apoptotic factor (Bax, Bcl-2, and Cleaved-Caspase3) protein expressions in LPS-induced IEC-6 cells after siTRPM7 transfection. ** means *P*<0.01 vs control group, ## means *P*<0.01 vs si-NC group

To verify whether TRPM7 participated in LPS-induced IEC-6 cell injury, the cell viability and apoptosis of LPS-induced IEC-6 cells were analyzed after transfection with siTRPM7-1 and siTRPM7-2. The results indicated that the transfection of siTRPM7s conspicuously reduced the mRNA and protein expressions of TRPM7 in LPS-induced IEC-6 cells (Figures 2A-2B) (*P*<0.01). Afterward, TRPM7 silencing promoted cell viability, while decreasing apoptotic rate, Bax/Bcl-2 ratio, and cleaved caspase 3 expression in IEC-6 cells after LPS treatment (Figures 2C-2G) (*P*<0.01).

***Knockdown of TRPM7 attenuated the inﬂammatory response in LPS-induced IEC-6 cells***

**Figure 3:**
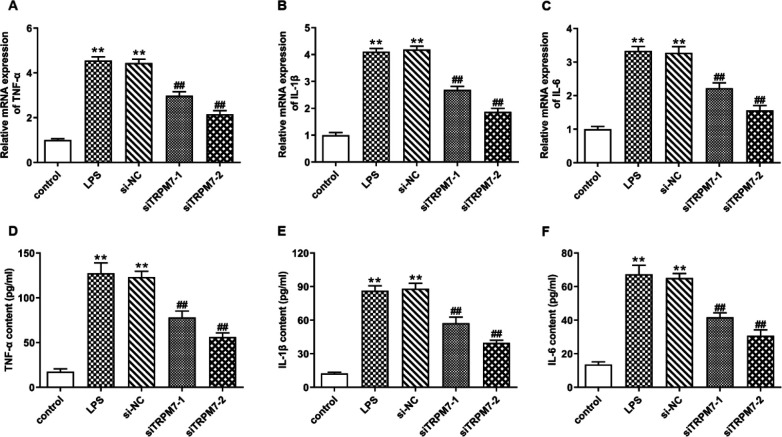
Knockdown of TRPM7 attenuated the inﬂammatory response in lipopolysaccharide (LPS)-induced IEC-6 cells. IEC-6 cells were transfected with the synthetic TRPM7 small interfering RNAs or si-NC for 48 hr before LPS treatment, with non-treated IEC-6 cells as control. (A-C) mRNA expressions of pro-inflammatory factors, TNF-α, IL-1β, and IL-6, in LPS-induced IEC-6 cells were detected by qRT-PCR. (D-F) Production of TNF-α, IL-1β, and IL-6 in the culture medium of LPS-induced IEC-6 cells was detected by ELISA. ** means *P*<0.01 vs control group, ## means *P*<0.01 vs si-NC group

The potential role of TRPM7 in LPS-induced inﬂammation in IEC-6 cells was further investigated. The results of qRT-PCR demonstrated that LPS significantly increased the mRNA expressions of TNF-α, IL-1β, and IL-6, while TRPM7 silencing showed an inhibitory effect (Figures 3A-3C) (*P*<0.01). In addition, the secretions of TNF-α, IL-1β, and IL-6 in the culture medium of IEC-6 cells showed an increase after LPS treatment, whereas they showed a decrease after siTRPM7 transfection before LPS treatment (Figures 3D-3F) (*P*<0.01).

***TRPM7 modulated TLR4/NF-κB and MEK/ERK pathways in LPS-induced IEC-6 cells***

**Figure 4:**
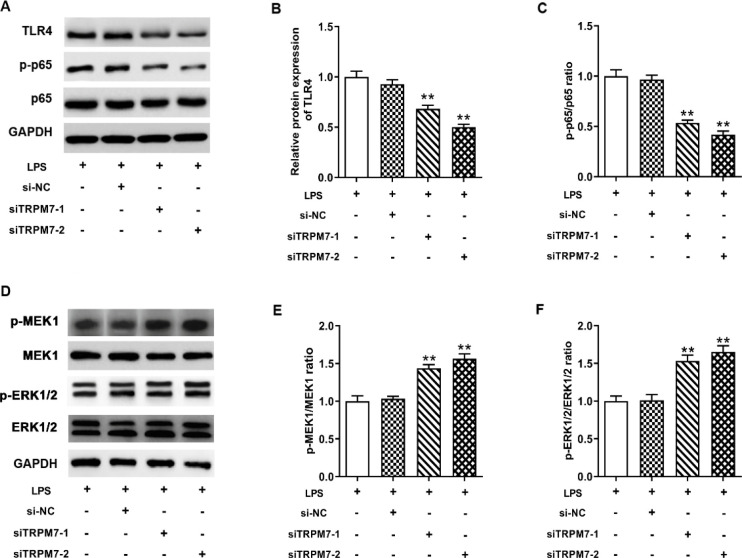
TRPM7 modulated TLR4/NF-κB and MEK/ERK pathways in lipopolysaccharide (LPS)-induced IEC-6 cells. IEC-6 cells were transfected with TRPM7 small interfering RNAs or si-NC before LPS stimulation. (A-C) Western blotting was used to measure the protein expressions of TLR4, and total and p-p65 in LPS-induced IEC-6 cells. (D-F) Western blotting was used to measure the protein expressions of total and p-MEK1, and total and p-ERK1/2 in LPS-induced IEC-6 cells. The non-transfected IEC-6 cells performed as a control. ** means *P*<0.01 vs LPS group

In order to explore the potential regulatory mechanism of TRPM7 in LPS-induced IEC-6 cells, we evaluated whether the TLR4/NF-κB and MEK/ERK pathways were involved in the regulation of TRPM7 on LPS-induced IEC-6 cell injury. It is reported that TRPM7 regulated TLR4/NF-κB and MEK/ERK pathways in various cells (25, 26). As shown in Figures 4A-4C, the protein expressions of TLR4 and p-p65/p65 ratio in IEC-6 cells were decreased by TRPM7 silencing (*P*<0.01). Inversely, the p-MEK1/MEK1 ratio and p-ERK1/2/ERK1/2 ratio were enhanced by siTRPM7s transfection (Figures 4D-4F) (*P*<0.01).

***Overexpression of TLR4 and inhibition of MEK attenuated the effects of siTRPM7 on LPS-induced apoptosis and inﬂammatory response***

**Figure 5:**
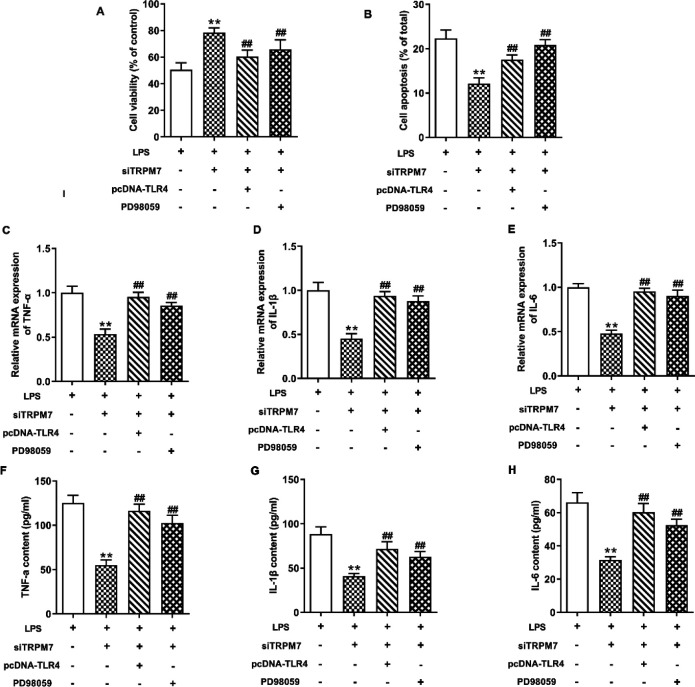
Overexpression of TLR4 and inhibition of MEK attenuated the effects of siTRPM7 on lipopolysaccharide (LPS)-induced apoptosis and inﬂammatory response. IEC-6 cells were treated with siTRPM7 and/or overexpression plasmids of TLR4 (pcDNA-TLR4), and PD98059 (a MEK-specific inhibitor) before LPS stimulation. (A) Cell viability was measured by a CCK-8 assay. (B) Cell apoptosis rate was detected by flow cytometry analysis. (C-E) mRNA expressions of TNF-α, IL-1β, and IL-6 were determined by qRT-PCR. (F-H) production of TNF-α, IL-1β, and IL-6 was measured by ELISA. ** means *P*<0.01 vs LPS group, ## means *P*<0.01 vs siTRPM7 group

We further validated the role of TLR4/NF-κB and MEK/ERK pathways in the regulation of TRPM7 on LPS-induced apoptosis and inflammation in IEC-6 cells. As shown in Figures 5A-5B, treatment with pcDNA-TLR4 or PD98059 decreased viability and increased the apoptosis rate of LPS-induced IEC-6 cells after siTRPM7 transfection (*P*<0.01). In addition, pcDNA-TLR4 and PD98059 attenuated the effects of TRPM7 silencing on mRNA expressions and secretion levels of TNF-α, IL-1β, and IL-6 in LPS-induced IEC-6 cells (Figures 5C-5H) (*P*<0.01).

## Discussion

NEC is a serious inflammatory bowel disease with high morbidity and fatality rates in newborns (3). In previous studies, NEC can be induced by LPS *in vitro*. LPS is one of the most abundant pro-inflammatory stimuli in the gastrointestinal tract (24). In this study, IEC cells were stimulated with LPS to establish the *in vitro* NEC cell model. Scientists have proven that inflammation and cell apoptosis were the main pathologies in the development of NEC (27). Consistent with a previous study (6, 24), LPS treatment inhibited viability and promoted apoptosis and inflammation of IEC-6 cells. Moreover, a recent study suggests that the progression of NEC was hindered via inhibiting apoptosis and inflammation of LPS-induced IEC-6 cells (28). Yuan *et al.* also found that TNF-α blocked the development of NEC via regulating LPS-mediated decreased viability and increased apoptosis of IEC-6 cells (24). In our study, TRPM7 was up-regulated in intestinal tissues of NEC patients and LPS-induced IEC-6 cells. Knockdown of TRPM7 suppressed cell apoptosis and inflammatory response in LPS-induced IEC-6 cells.

TRPM7 distributes in various tissues and organs, as well as participating in the regulation of cell growth and proliferation (29, 30). Mittermeier *et al. *suggested that TRPM7 regulated intestinal mineral absorption in mice (31). Besides, TRPM7 reportedly regulated intestinal motility by inhibiting pacemaker potentials in interstitial cells of Cajal in mice (12, 13). Importantly, it has been reported that TRPM7 was overexpressed in human inflammatory bowel disease (IBD) and colorectal cancer (14). Based on these studies, we studied the role of TRPM7 in NEC. We found that TRPM7 expression was up-regulated in NEC tissues and LPS-treated IEC-6 cells. Knockdown of TRPM7 attenuated LPS-induced cell apoptosis and inflammatory response in IEC-6 cells. Moreover, the role of TRPM7 in the regulation of inflammation response in various diseases has been reported. It is reported that Tanshinone IIA inhibited LPS-induced pro-inflammatory factors in acute lung injury by suppressing TRPM7 expression (15). Down-regulation of TRPM7 reduced inflammation of rat bone marrow-derived mast cells (32). In cardiovascular diseases, TRPM7 also has an anti-inflammatory effect (16).

Evidence implies that TRPM7 regulates the endocytosis of TLR4 and nuclear translocation of NF-κB induced by LPS in macrophages (33). Besides, TRPM7 was involved in the LPS-induced migration of endothelial cells via the TLR4/NF-κB signaling pathway (34). Similarly, our data showed that knockdown of TRPM7 blocked the TLR4/NF-κB pathway activation. Importantly, it has been reported that the TLR4/NF-κB pathway contributed to the pathogenesis of NEC, which was consistent with our findings (19). After LPS stimulation, up-regulation of TLR4 triggers a series of transmembrane conduction responses that activates NF-κB ultimately leading to the release of downstream inflammatory cytokines that induces intestinal tissue damage (18, 35). Moreover, growing experimental studies have demonstrated that the inhibition of the TLR4/NF-κB pathway significantly inhibited NEC-induced intestinal cell apoptosis and inflammatory response (17, 28).

Furthermore, involvement of TRPM7 in the MEK/ERK pathway has been observed in a variety of cells (25, 26). For example, TRPM7 inhibition promotes cell proliferation by activating MEK/ERK pathway in pulmonary artery smooth muscle cells, vascular smooth muscle cells, and aortic vascular smooth muscle cells (20, 26, 36). The present study also revealed that knockdown of TRPM7 enhanced MEK/ERK pathway activation in LPS-induced IEC-6 cells. On the one hand, several studies indicate that inhibition of the MEK/ERK pathway attenuates the progression of ulcerative colitis (21). On the other hand, the function of the MEK/ERK pathway in the protection of IEC cells from apoptosis in Crohn’s disease colitis has been proven (23). Recently, studies have shown that activating the ERK pathway reduced apoptosis and inflammation of the NEC rat experimental model and LPS induced NEC cell model (22, 37). What is more, our data suggested that knockdown of TRPM7 reduced LPS-induced apoptosis and inflammation in IEC6 cells by activating the MEK/ERK pathway.

## Conclusion

TRPM7 was overexpressed in NEC tissues and LPS-induced NEC cell model. In addition, knockdown of TRPM7 attenuated LPS-induced apoptosis and inflammation of IEC6 cells by inactivating the TLR4/NF-κB pathway and activating MEK/ERK pathway, which suggested that TRPM7 might serve as a potential therapeutic target for NEC prevention and treatment.

## Authors’ Contributions

QW Conceived and designed the work; JL and BL Provided data acquisition and analysis; JH, QZ, and XZ Interpreted the data for the work; LA Drafted the work and revised it critically for important intellectual content.

## Availability of Data and Material

The data used to support the findings of this study are available from the corresponding author upon request.

## Conflicts of Interest

The authors have no relevant financial or non-financial interests to disclose.
